# Comparison of Sleeve Gastrectomy vs Intensive Lifestyle Modification in Patients With a BMI of 30 to Less Than 35

**DOI:** 10.1001/jamanetworkopen.2022.23927

**Published:** 2022-07-27

**Authors:** Erik Stenberg, Gustaf Bruze, Johan Sundström, Claude Marcus, Ingmar Näslund, Johan Ottosson, Martin Neovius

**Affiliations:** 1 Department of Surgery, Faculty of Medicine and Health, Örebro University, Örebro, Sweden; 2 Clinical Epidemiology Division, Department of Medicine, Solna, Karolinska Institutet, Stockholm, Sweden; 3 Department of Medical Sciences, Uppsala University, Uppsala, Sweden; 4 Department of Clinical Science, Intervention and Technology, Karolinska Institutet, Stockholm, Sweden

## Abstract

**Importance:**

There is a lack of studies evaluating sleeve gastrectomy compared with intensive lifestyle treatment in patients with class 1 obesity (body mass index [BMI] 30 to <35 [calculated as weight in kilograms divided by height in meters squared]).

**Objective:**

To compare outcomes and safety of sleeve gastrectomy compared with intensive nonoperative obesity treatment in patients with class 1 obesity.

**Design, Setting, and Participants:**

This matched, nationwide cohort study included patients with class 1 obesity who underwent a sleeve gastrectomy or intensive lifestyle treatment between January 1, 2012, and December 31, 2017, and who were registered in the Scandinavian Obesity Surgery Registry or the Itrim health database. Participants with class 1 obesity were matched 1:2 using a propensity score including age, sex, BMI, treatment year, education level, income, cardiovascular disease, and use of antibiotic drugs, antidepressants, and anxiolytics.

**Interventions:**

Sleeve gastrectomy or intensive lifestyle treatment.

**Main Outcomes and Measures:**

Outcomes included weight loss after intervention, changes in metabolic comorbidities, substance use disorders, self-harm, and major cardiovascular events retrieved from the National Patient Register, Prescribed Drug Register, and Cause of Death Register as well as the Scandinavian Obesity Surgery Registry and the Itrim health database. Data were analyzed from December 1, 2021 until May 31, 2022.

**Results:**

The study included 1216 surgery patients and 2432 lifestyle participants with similar mean (SD) BMI (32.8 [1.4] vs 32.9 [1.4]), mean (SD) age (42.4 [9.7] vs 42.6 [12.7] years), and sex (1091 [89.7%] vs 2191 [90.1%] women). Surgery patients had greater 1-year weight loss compared with controls (22.9 kg vs 11.9 kg; mean difference, 10.7 kg; 95% CI, 10.0-11.5 kg; *P* < .001). Over a median follow-up of 5.1 years (IQR, 3.9-6.2 years), surgery patients had a lower risk of incident use of diabetes drugs (59.7 vs 100.4 events per 10 000 person-years; hazard ratio [HR], 0.60; 95% CI, 0.39-0.92; *P* = .02) and greater 2-year diabetes drug remission (48.4% vs 22.0%; risk difference 26.4%; 95% CI, 11.7%-41.0%; *P* < .001), but higher risk for substance use disorder (94 vs 50 events per 10 000 person-years; HR, 1.86; 95% CI, 1.30-2.67; *P* < .001) and self-harm (45 vs 25 events per 10 000 person-years; HR, 1.81; 95% CI, 1.09-3.01; *P* = .02). No between-group difference in occurrence of major cardiovascular events was observed (23.4 vs 24.8 events per 10 000 person-years; HR, 0.96; 95% CI, 0.49-1.91; *P* = .92).

**Conclusions and Relevance:**

In this cohort study, compared with intensive nonoperative obesity treatment, sleeve gastrectomy in patients with class 1 obesity was associated with greater weight loss, diabetes prevention, and diabetes remission but a higher incidence of substance use disorder and self-harm.

## Key Points

**Question:**

What are the outcomes and safety of sleeve gastrectomy compared with intensive nonoperative obesity treatment in patients with class 1 obesity?

**Findings:**

In this matched cohort study including 1216 patients who underwent sleeve gastrectomy and 2432 controls who underwent intensive lifestyle treatment, surgery was associated with greater weight loss after the intervention, lower incidence of diabetes, and a higher rate of pharmacological diabetes remission, but a higher risk for self-harm and substance use disorder.

**Meaning:**

These findings suggest that sleeve gastrectomy may be a treatment option in selected patients with metabolic comorbidity and class 1 obesity, although careful preoperative evaluation and optimization of risk factors along with close support and follow-up after surgery remain important, in particular for patients at increased risk for psychiatric comorbidities, including substance use disorder and self-harm.

## Teaser

This cohort study evaluates the outcomes and safety associated with sleeve gastrectomy compared with intensive nonoperative obesity treatment in patients with class 1 obesity in Sweden.

## Introduction

The current body mass index (BMI)–based indications for bariatric surgery with a BMI of 40 or greater alone or BMI of 35 or greater (BMI calculated as weight in kilograms divided by height in meters squared) and at least 1 obesity-related comorbidity were established in a consensus meeting in 1991.^1^ Inspired by the metabolic benefits for patients with severe obesity, clinical trials comparing gastric bypass (and, in 1 study, gastric banding) with nonoperative supportive care including patients with class 1 obesity (BMI 30 to <35) have reported beneficial results for patients with type 2 diabetes or hypertension.^2,3,4,5,6,7,8^

Although the gastric bypass procedure remains the criterion standard of surgical treatment for obesity, sleeve gastrectomy has increased in popularity and is now the most common bariatric surgical procedure worldwide.^9^ The medium- and long-term results for sleeve gastrectomy are reported to compare well to those of gastric bypass for patients with a BMI of 35 or greater.^10^ By being perceived as a technically less challenging operation than the gastric bypass procedure, with a shorter operation time and a general view that short-term complications may be lower, sleeve gastrectomy is often considered for patients with class 1 obesity despite the lack of strong scientific support.^11^ While good weight-loss results have been reported from case series, there is a lack of large controlled studies addressing objective effectiveness and safety outcomes beyond 1 or 2 years compared with actively treated control individuals with class 1 obesity.^12^

The aim of this study was to evaluate outcomes and safety for up to 8 years of follow-up comparing sleeve gastrectomy with active nonoperative obesity treatment in patients with class 1 obesity.

## Methods

This nationwide matched cohort study with prospectively registered data includes individuals from the Scandinavian Obesity Surgery Registry, covering more than 97% of all patients treated with bariatric surgery in Sweden from 2012 through 2017^13^; and the Itrim health database, with individuals undergoing a 1-year structured intensive lifestyle modification program.^14,15^ All analyses were conducted on deidentified data, and the study was approved by the regional ethics review board in Stockholm, Sweden, with the requirement for informed consent being waived. This study followed the Strengthening the Reporting of Observational Studies in Epidemiology (STROBE) reporting guideline.

### Inclusion and Exclusion Criteria

The study population was restricted to individuals 18 years or older and with a BMI at screening between 30 and less than 35. Only primary sleeve gastrectomy procedures were considered, excluding revision procedures. Because sleeve gastrectomy was uncommon in Sweden before 2012 and we required a minimum of 3 years of register-based follow-up, the study population was restricted to those who underwent operation or initiated lifestyle modification between January 1, 2012, and December 31, 2017.

### Interventions

#### Sleeve Gastrectomy

The intervention of choice was mainly based on patient preference. Patients in the sleeve gastrectomy cohort were treated at any of the centers performing bariatric surgery in Sweden during the study period. The majority of patients underwent a short, very-low-calorie or low-calorie diet as a preparation for surgery, followed by a sleeve gastrectomy routinely using a 32-36 French bougie, starting the resection at no more than 5 cm from the pylorus and ending 1 cm from the angle of His. Surgery patients were followed up clinically at the surgery department at approximately 6 weeks, 1 year, and 2 years after operation.

#### Intensive Lifestyle Modification

Individuals underwent a 3-month dietary weight-loss phase facilitated by a low- or very-low-calorie diet followed by a 9-month weight maintenance phase.^14^ The choice of a low-calorie or very-low-calorie diet was based on baseline BMI, personal preference, and contraindication status. After the weight-loss phase, patients entered a 9-month weight maintenance program including exercise (circuit training at a center 2-3 times per week for 30-45 minutes, and pedometer use to encourage walking) and dietary advice. Behavioral changes were facilitated by a structured program including 20 group sessions lasting 1 hour and face-to-face counselling sessions. The sessions covered specific topics, such as health benefits of weight loss, healthy eating strategies, finding realistic eating and exercise routines, and stress management.^14^

Intensive lifestyle participants were selected as controls because they also had obesity, they intended to lose weight, and their treatment led to more weight loss^15^ than most other common control treatments.^14,16^

### Outcomes and Follow-up

Outcomes included data from the National Patient Register, Prescribed Drug Register, and Cause of Death Register until October 30, 2020, as well as weight loss data from the Scandinavian Obesity Surgery Registry and Itrim. Outcomes included 1- and 2-year weight-loss, changes in diabetes and hypertension (incident drug-treated diabetes or hypertension; use of insulins and noninsulins or antihypertensives), any major cardiovascular event (including unstable angina (*International Statistical Classification of Diseases and Related Health Problems, Tenth Revision *code I20.0), myocardial infarction (I21-I22), acute cerebrovascular event (I60, I61, I63, and I64), fatal cardiovascular event (cause of death I01-I78, excluding I30), or unattended sudden cardiac death (R96.0, R96.1, R98, and R99), and all-cause mortality.

Safety outcomes included early postoperative complications (defined as any occurrence of a complication, a serious postoperative complication defined as Clavien-Dindo 3b or higher, or readmission into inpatient care within 30 days of index surgery) and revision surgery for the surgical cohort, use of gastric acid–suppressing agents, and psychiatric disorders (substance use disorder, suicide or self-harm, use of antidepressants, and use of anxiolytics) for both groups. For the early postoperative complications, patients with a BMI of 35 or greater who underwent sleeve gastrectomy in Sweden during the same period were included for comparison.

For outcomes including inpatient or hospital-based outpatient care, data were retrieved from the National Patient Register. For drug use outcomes, data on filled prescriptions were retrieved from the Prescribed Drug Register, and individuals were classified as users or nonusers of drugs within 6-month intervals relative to the time of treatment. Individuals were followed from 5 years before treatment initiation (when applicable) until death, emigration, or end of follow-up (maximum 8.9 years). Data on death and emigration were retrieved from the Swedish Total Population Register,^17^ and suicide information was retrieved from the Causes of Death Register.

### Covariates

Baseline weight and height were used to calculate BMI (weight in kilograms divided by height in meters squared) with consistent registrations in the databases. Data on age, sex, education, income, and marital status were collected from the Longitudinal Integration Database for Health Insurance and Labor Market studies^18^ and the Total Population Register^17^ from Statistics Sweden. Data on hospital visits for substance use disorder and self-harm, as well as for cardiovascular disease, were obtained from the National Patient Register (nationwide inpatient data from 1987; hospital-based outpatient data from January 1, 2001). Filled prescriptions for diabetes drugs, antihypertensive drugs, lipid-modifying drugs, gastric acid–suppressing agents, antidepressants, and anxiolytics were retrieved from the Prescribed Drug Register (*International Classification of Diseases* and *Anatomical Therapeutic Chemical* codes used are provided in eTable 1 in the Supplement).

### Statistical Analysis

Data were analyzed between December 1, 2021, and May 31, 2022. Propensity score matching was used to make the surgery and the intensive lifestyle groups comparable. Because there were more eligible individuals in the intensive lifestyle group, each surgery patient was matched to 2 comparators. The propensity score was estimated from a logistic regression model and defined as the estimated probability of receiving either surgical or intensive lifestyle treatment.

The covariates used in the logistic model were age (continuous), sex, BMI (continuous), intervention year, education level (<10, 10-12, or >12 years), disposable income (continuous), physical comorbidity (diabetes drug treatment, cardiovascular disease), and psychiatric disorders (use of antidepressants or use of anxiolytics; detailed covariate description provided in eTable 2 in the Supplement). All variables except for BMI were retrieved from national registers, resulting in little to no missing data (0.5%-0.6% for education; 0.1%-0.2% for income; individuals with missing education or income data were excluded) (eFigure 1 in the Supplement).

In the matched cohort, we evaluated 1- and 2-year weight loss using linear regression, adjusting for weight at baseline. We also computed the mean adjusted difference between the surgery and intensive lifestyle groups with respect to binary indicators for filled prescriptions within 6-month intervals relative to the time of treatment using linear regression and adjusting for age, sex, and education level. Time-to-event analysis was performed using Cox regression. The proportional hazard assumption was tested using an interaction term for surgery treatment and time. Time to event data were illustrated using Kaplan-Meier curves.

Analyses were performed using SAS, version 9.4 (SAS Institute Inc) and Stata, version 14 (StataCorp LLC). Statistical significance was defined as a 2-sided *P* value of less than .05 in combination with relevant effect size.

## Results

During the inclusion period, 1216 sleeve patients were identified and matched to 2432 controls from the lifestyle modification group (eFigure 1 in the Supplement). The matching generated 2 cohorts that were well balanced with a standardized difference of less than 0.1 for all baseline characteristics, including a mean (SD) BMI of 32.8 (1.4) and mean (SD) age of 42.4 (9.7) years in the surgical group and a mean (SD) BMI of 32.9 (1.4) and mean (SD) age of 42.6 (12.7) years in the lifestyle modification group. The majority of patients in both groups were women (1091 [89.7%] in the sleeve gastrectomy group and 2191 [90.1%] in the lifestyle modification group) (Table). Median follow-up time was 5.1 years (IQR, 3.9-6.2 years) in the sleeve group and 5.1 years (IQR, 4.0-6.2 years) in the lifestyle modification group.

**Table.** Participant Characteristics by Treatment Group

Characteristic	Participants, No. (%)		Standardized difference
	Sleeve gastrectomy (n = 1216)	Intensive lifestyle modification (n = 2432)	
Age, mean (SD), y	42.4 (9.7)	42.6 (12.7)	0.019
Sex			
Women	1091 (89.7)	2191 (90.1)	0.012
Men	125 (10.3)	241 (9.9)	0.012
BMI at screening, mean (SD)	32.8 (1.4)	32.9 (1.4)	0.015
Socioeconomic factors			
Educational level			
Primary school	118 (9.7)	225 (9.3)	0.015
High school	650 (53.5)	1306 (53.7)	0.005
University	448 (36.8)	901 (37.0)	0.004
Annual income, mean (SD), $	31 400 (23 800)	30 900 (32 300)	0.015
Physical comorbidity			
Diabetes drugs	94 (7.7)	165 (6.8)	0.037
Insulins	33 (2.7)	43 (1.8)	0.066
Noninsulins	86 (7.1)	150 (6.2)	0.037
Antihypertensive therapy	245 (20.1)	473 (19.4)	0.018
Lipid-lowering therapy	115 (9.5)	222 (9.1)	0.011
Circulatory disease^a^	227 (18.7)	462 (19.0)	0.008
Cardiovascular comorbidity^b^	32 (2.6)	77 (3.2)	0.031
Cerebrovascular disease^c^	6 (0.5)	26 (1.1)	0.062
Psychiatric comorbidity			
Psychiatric hospitalization	99 (8.1)	175 (7.2)	0.036
Psychiatric outpatient care	281 (23.1)	482 (19.8)	0.081
Antidepressants	578 (47.5)	1171 (48.1)	0.012
Anxiolytics	424 (34.9)	836 (34.4)	0.011
Substance use disorder	60 (4.9)	119 (4.9)	0.002

Abbreviation: BMI, body mass index (calculated as weight in kilograms divided by height in meters squared).

^a^ Chapter I in *International Statistical Classification of Diseases and Related Health Problems, Tenth Revision *(*ICD-10*).

^b^ Defined as a previous diagnosis of heart failure (*ICD-10*: I50), acute myocardial infarction or angina pectoris (*ICD-10*: I21-I22), atrial fibrillation, flutter or other tachycardia (*ICD-10*: I47-I48).

^c^ Defined as a previous cerebrovascular event (*ICD-10*: I60, I61, I63, or I64).

### Weight Loss

**Figure 1.**
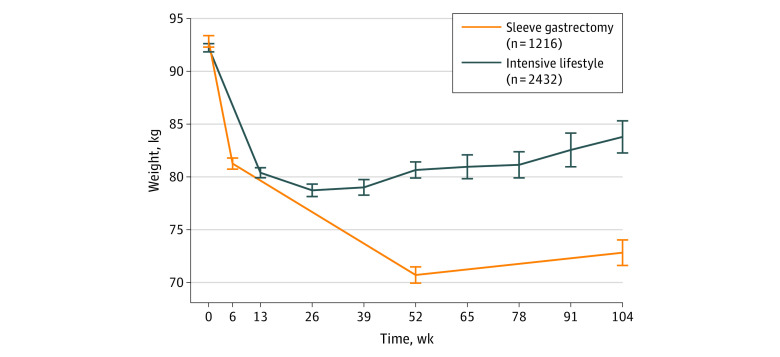
Weight Development Up to 2 Years After Intervention

At baseline, the sleeve group had a mean (SD) body weight of 92.8 (9.6) kg compared with 92.2 (9.8) kg in the lifestyle group (standardized difference, 0.062). Patients in the sleeve group experienced higher weight loss at 1 year compared with the lifestyle group (22.9 kg [95% CI, 22.3-23.4 kg] vs 11.9 kg [95% CI, 11.4-12.4 kg]; mean difference, 10.7 kg [95% CI, 10.0-11.5 kg]; *P* < .001) and at 2 years (21.0 kg [95% CI, 20.2-21.9 kg] vs 8.8 kg [95% CI, 7.6-9.9 kg]; mean difference, 12.0 kg [95% CI, 10.6-13.4 kg]; *P* < .001) (Figure 1). In terms of percentage of total weight loss, these values corresponded to 24.4% of total weight loss (95% CI, 23.8%-25.0%) in the surgery group vs 12.8% of total weight loss (95% CI, 12.2%-13.3%) in the lifestyle group at 1 year (mean difference, 11.6% [95% CI, 10.8%-12.4%]; *P* < .001), and 22.4% (95% CI, 21.5%-23.3%) vs 9.4% (95% CI, 8.2%-10.6%) at 2 years (mean difference, 13.0% [95% CI, 11.6%-14.5%]; *P* < .001).

### Medication and Cardiovascular Outcomes

**Figure 2.**
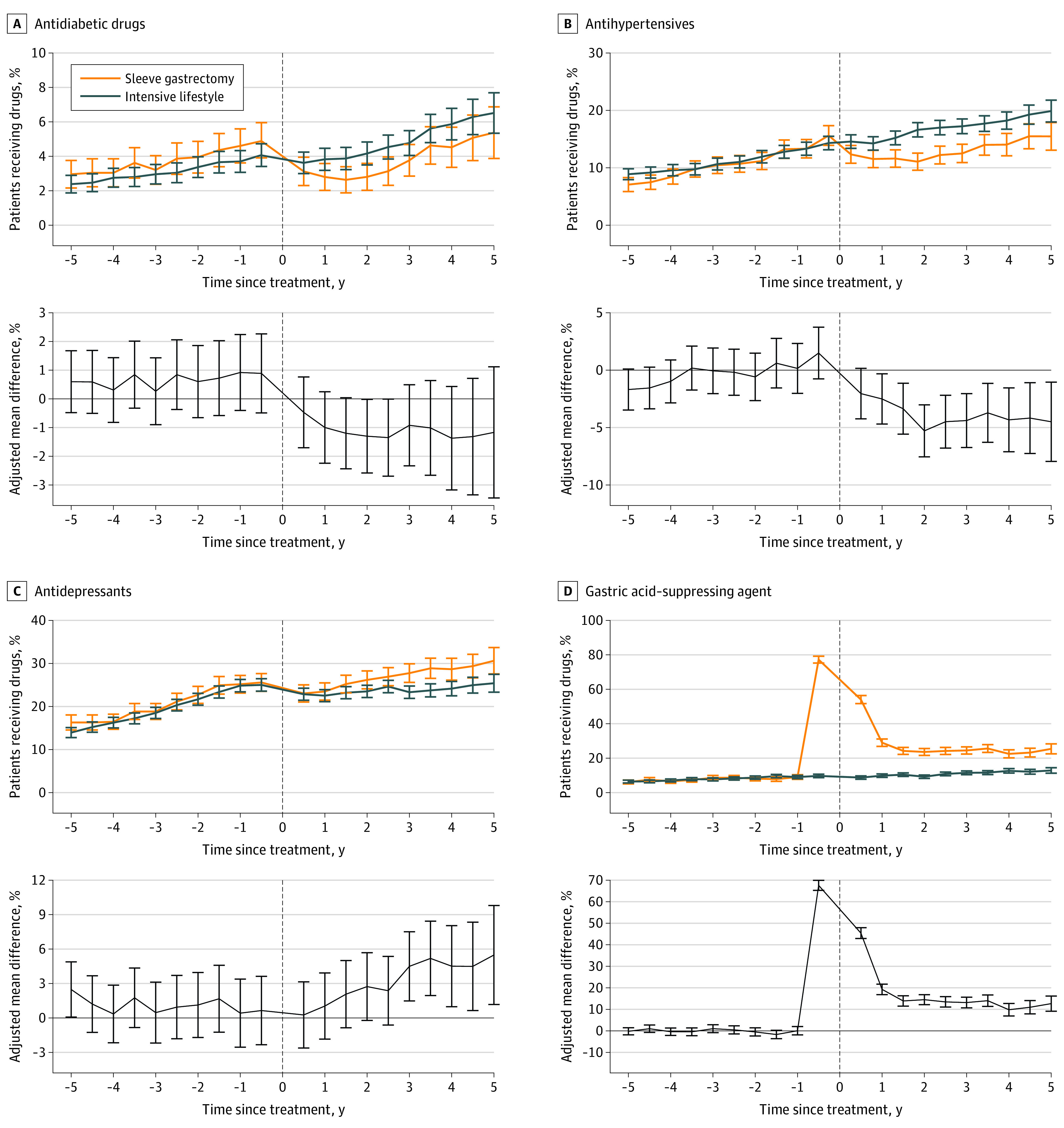
Use of Antidiabetic Drugs, Antihypertensives, Antidepressants, and Gastric Acid–Suppressing Agents

**Figure 3.**
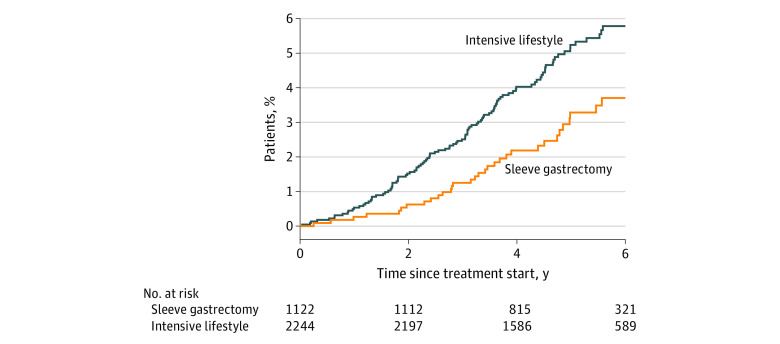
Incident Use Of Diabetes Drugs Among Participants Without Diabetes Drug Prescriptions Before Intervention

The sleeve group experienced a reduction in pharmacological treatment of diabetes, with lower diabetes drug use than the lifestyle group during 5 years of follow-up, although the differences were not statistically significant for each individual year (Figure 2; eFigure 3 in the Supplement). In participants without diabetes drug prescriptions before intervention, the risk of incident use was lower in the sleeve group than in the lifestyle group (59.7 vs 100.4 events per 10 000 person-years; HR, 0.60; 95% CI, 0.39-0.92; *P* = .02) (Figure 3). In the subgroup with drug-treated diabetes at baseline, the 2-year remission rate was 48.4% in the surgery group compared with 22.0% in the lifestyle group (risk difference, 26.4%; 95% CI, 11.7%-41.0%; *P* < .001).

The sleeve group also experienced a reduction in antihypertensive treatment with consistently lower use of antihypertensive drugs than the lifestyle group (Figure 2). While the lifestyle group increased antihypertensive drug use from around 15% to 20% from baseline to the 5-year follow-up, the sleeve group remained around 15% use. No differences in the use of lipid-lowering drugs were observed (Figure 2, eFigure 2 in the Supplement).

**Figure 4.**
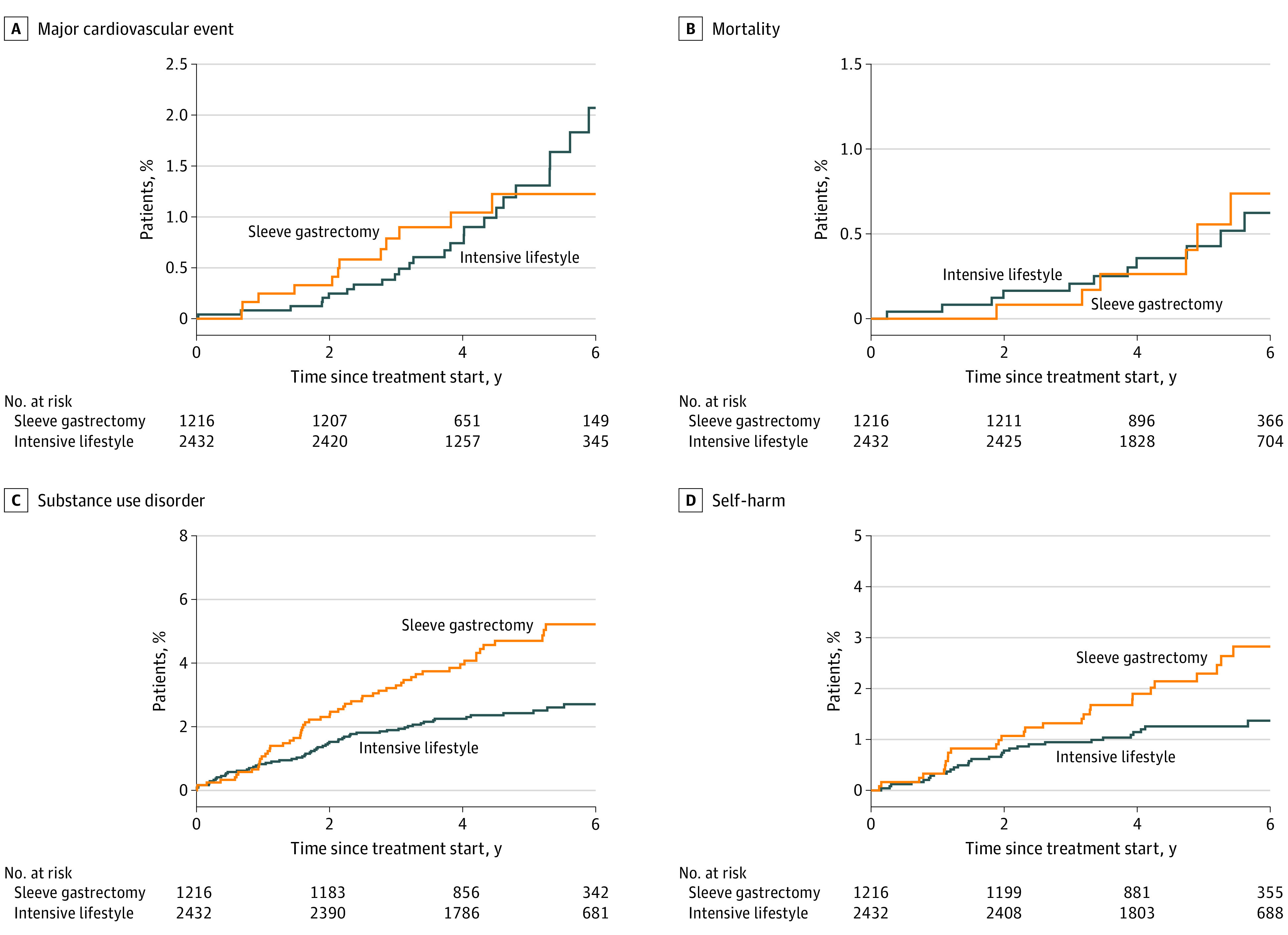
Risk of Major Cardiovascular Event, Mortality, Substance Use Disorder, and Self-harm After Intervention

The sleeve and lifestyle groups experienced similar risks for major cardiovascular event (23.4 vs 24.8 events per 10 000 person-years; HR, 0.96; 95% CI, 0.49-1.91; *P* = .92) and all-cause mortality (9.6 vs 11.8 events per 10 000 person-years; HR, 0.87; 95% CI, 0.34-2.23; *P* = .76) (Figure 4).

### Safety Outcomes

Median duration of surgery was 45 minutes (IQR, 35-56 minutes), and median length of stay 2 days (IQR, 1-2 days). During the 30 days after surgery, 33 of 1216 sleeve patients (2.7%) were readmitted to hospital (eTable 3 in the Supplement). Clinical follow-up data at day 30 were available for 1106 of 1216 patients (90.9%), 56 (5.1%) of whom were reported to have any complication, including 16 (1.4%) serious complications (eTable 3 in the Supplement). Compared with sleeve gastrectomies performed in patients with a BMI of 35 or greater, patients with a BMI between 30 and less than 35 had a lower readmission risk (2.7% vs 3.8%) and a similar risk of any complication (5.1% vs 5.6%) as well as serious complications (1.4% vs 1.9%) (eTable 3 in the Supplement).

During follow-up, 22 patients (1.8%) underwent revision surgery. Weight regain (n = 12) and gastroesophageal reflux (n = 10) were the most common contributing factors for revision, followed by malnutrition or stenosis (n = 4) and surgical complication manifesting as leakage (n = 2).

Owing to the high perioperative prophylactic use of gastric acid–suppressing agents, the use of these agents increased from approximately 10% to 80% around the time of surgery, while it remained flat in the lifestyle group (Figure 2). During the 5 years of follow-up, use of these agents remained at a higher level of approximately 15 percentage points in the sleeve group compared with the lifestyle group.

Use of antidepressants and anxiolytics was balanced between the groups before treatment and decreased during the first year after intervention; thereafter, the use of both drug types increased more in the sleeve group than the lifestyle group, resulting in greater use for most follow-up years (Figure 2, eFigure 2 in the Supplement).

The sleeve group also experienced higher rates of substance use disorder (94 vs 50 events per 10 000 person-years; HR, 1.86; 95% CI, 1.30-2.67; *P* < .001) and self-harm (45 vs 25 events per 10 000 person-years; HR, 1.81; 95% CI, 1.09-3.01; *P* = .02) (Figure 3). The majority of substance use disorders represented alcohol use disorders (69 vs 30 events per 10 000 person-years; HR, 2.24; 95% CI, 1.45-3.48; *P* < .001) (eFigure 4 in the Supplement).

## Discussion

In this cohort study, sleeve gastrectomy in patients with class 1 obesity was associated with greater weight loss, higher diabetes remission rates, and lower incidence of new-onset diabetes and hypertension compared with intensive lifestyle treatment. Perioperative complication rates were low, but an increased use of gastric acid–suppressing agents and an elevated incidence of substance use disorder and self-harm were seen after surgery compared with the intensive lifestyle intervention. No differences were observed for major cardiovascular events and all-cause mortality.

Sleeve gastrectomy has been reported to be safe with promising weight loss during short- to mid-term follow-up for patients with class 1 obesity,^12,19,20^ but to date there are no studies comparing these results with a matched control group. Despite the lack of scientific support, evidence from Roux-en-Y gastric bypass (RYGB) and gastric banding have encouraged surgeons to operate on patients with class 1 obesity with sleeve gastrectomy. While slightly lower weight loss and smaller changes in hypertension and dyslipidemia have been reported as compared with RYGB,^10^ the lower complication rates and shorter operation time make sleeve gastrectomy an appealing option for patients with class 1 obesity. The association of sleeve gastrectomy with improvement in metabolic comorbidities and weight loss reported for patients with a preoperative BMI greater than 35 was also reflected in the present study.^8,21^

The sleeve group experienced higher rates of diabetes remission (as defined by absence of pharmacological treatment) as well as fewer patients requiring antihypertensive medication compared with the intensive lifestyle group. In addition, new onset of diabetes was lower after surgery. Despite these improvements, no differences in occurrence of major cardiovascular events or mortality were seen. A reasonable explanation for this may be that both groups were on average healthier than patients included in many previous studies focusing mainly on those with more advanced disease.^21^ For a nonselected group, even with higher BMI, a longer duration of follow-up may also be necessary to detect such differences.^22^

Many of the problematic aspects of bariatric surgery reported for patients with higher BMI were also seen in the present study. One of the major concerns with sleeve gastrectomy is related to gastroesophageal reflux disease and the potential development of Barrett esophagus and risk of transformation into adenocarcinoma.^23,24^ Increased use of gastric acid–suppressing agents and a small albeit nonnegligible conversion rate (10 of 1216 patients) due to gastroesophageal reflux disease was seen after sleeve gastrectomy in this study, signaling increased gastroesophageal reflux disease after surgery. The long-term importance of this complication remains to be evaluated. Some studies have reported alarming numbers of Barrett esophagus among patients with these complications,^25^ whereas others have not.^26^

Substance use disorder, treatment for depression and anxiety, and health care encounters for self-harm were all increased in the surgery group compared with the intensive lifestyle treatment group. These problematic side effects have been reported earlier after gastric surgery for duodenal ulcer^27^ and later for RYGB.^28^ A combination of altered anatomy with hormonal differences, changed pharmacodynamics, and increased alcohol uptake, as well as psychosocial factors influencing relationships, frustration, and psychosocial maladaptation have been suggested as potential explanatory factors for these outcomes after RYGB.^29^ Less is known about these outcomes in association with sleeve gastrectomy. Previous studies have reported increased alcohol uptake after sleeve gastrectomy similar to after RYGB.^30^ Careful evaluation of patients before surgery, optimization of risk factors, and close support during the perioperative and postoperative periods remain important measures, in particular for patients at increased risk of these complications.

Overall, many outcomes (positive and negative) were very similar to those reported for patients with BMI greater than 35. Although randomized clinical trials are needed to confirm causation, the results of this study support the use of sleeve gastrectomy in the treatment of selected patients with class 1 obesity. The use of strict BMI cutoff values, as suggested in the often-used National Institutes of Health criteria from 1991, have indeed been questioned because of low sensitivity to identify excess adiposity.^31^ As indicated by the results of this study, sleeve gastrectomy may be an appropriate valid option for surgical treatment of patients with prediabetes, diabetes or hypertension, and class 1 obesity. The use of sleeve gastrectomy for patients with class 1 obesity without metabolic comorbidities remains questionable in light of the long-term side effects.

### Strengths and Limitations

This study is, to our knowledge, the first large study on sleeve gastrectomy in patients with class 1 obesity with a BMI-matched control group receiving a nonoperative intensive intervention, additionally matched on multiple physical, psychiatric, and socioeconomic variables. Major strengths include its nationwide design and prospectively collected outcome data with complete information about filled prescriptions and health care visits from 5 years before to up to 8 years after either sleeve gastrectomy or intensive lifestyle modification.

This study also has some limitations. Despite the matching, this remains an observational study and, as such, cannot evaluate causation. The 2 groups generally had higher socioeconomic status compared with the majority of patients who underwent bariatric surgery in Sweden. The groups also represent patients seeking either treatment option; thus, funding by indication cannot be ruled out, which could influence some of the secondary end points. Furthermore, the median follow-up was only 5 years, which is likely too short a timeframe to fully evaluate mortality and cardiovascular events, especially given the lower cardiovascular risk in the patient population with class 1 obesity compared with patients with higher BMI. Finally, psychiatric comorbidity was defined by pharmacological use or contacts in specialized care. Nonpharmacological treatment in private medicine would be missed, resulting in underestimation of psychiatric disorders. However, this limitation is likely to influence both groups to a similar extent and thus not introduce differential bias. Further studies using a randomized design are needed to confirm causation.

## Conclusions

In this cohort study, compared with intensive, nonoperative obesity treatment, sleeve gastrectomy in patients with class 1 obesity was associated with greater weight loss and higher remission as well as prevention of diabetes and hypertension; however, it was also associated with higher use of gastric acid–suppressing agents and a higher incidence of substance use disorder and self-harm. These findings suggest that sleeve gastrectomy may be an appropriate treatment option in selected patients with metabolic comorbidity and class 1 obesity.

## Notes

Supplement. **eFigure 1.** Flowchart **eFigure 2.** Use of lipid-lowering drugs and anxiolytics. Red representing surgery group, blue representing intensive lifestyle modification group **eFigure 3.** Diabetes medication before and after treatment for patients with diabetes drug treatment before intervention **eFigure 4.** Alcohol use disorders after intervention for patients receiving sleeve gastrectomy or intensive lifestyle treatment **eTable 1.** Outcome definitions **eTable 2.** Description and definitions of propensity score variables **eTable 3.** Surgical complications within 30 days following sleeve gastrectomy
