# Supplementary material for: Comparison of Sleeve Gastrectomy vs Intensive Lifestyle Modification in Patients With a BMI of 30 to Less Than 35
Source: JAMA Netw Open. 2022 Jul 27;5(7):e2223927. doi: 10.1001/jamanetworkopen.2022.23927 (PMC9331100; doi:10.1001/jamanetworkopen.2022.23927)
Supplement: Supplement. — eFigure 1. Flowchart eFigure 2. Use of lipid-lowering drugs and anxiolytics. Red representing surgery group, blue representing intensive lifestyle modification group eFigure 3. Diabetes medication before and after treatment for patients with diabetes drug treatment before intervention eFigure 4. Alcohol use disorders after intervention for patients receiving sleeve gastrectomy or intensive lifestyle treatment eTable 1. Outcome definitions eTable 2. Description and definitions of propensity score variables eTable 3. Surgical complications within 30 days following sleeve gastrectomy [file jamanetwopen-e2223927-s001.pdf]

Supplemental Online Content

Stenberg E, Bruze G, Sundström J, et al. Comparison of sleeve gastrectomy vs intensive lifestyle modification in patients with a BMI of 30 to less than 35. *JAMA Netw Open*. 2022;5(7):e2223927. doi:10.1001/jamanetworkopen.2022.23927

eFigure 1. Flowchart

eFigure 2. Use of lipid-lowering drugs and anxiolytics. Red representing surgery group, blue representing intensive lifestyle modification group

eFigure 3. Diabetes medication before and after treatment for patients with diabetes drug treatment before intervention

eFigure 4. Alcohol use disorders after intervention for patients receiving sleeve gastrectomy or intensive lifestyle treatment

eTable 1. Outcome definitions

eTable 2. Description and definitions of propensity score variables

eTable 3. Surgical complications within 30 days following sleeve gastrectomy

This supplemental material has been provided by the authors to give readers additional information about their work.

© 2022 Stenberg E et al. *JAMA Network Open.*

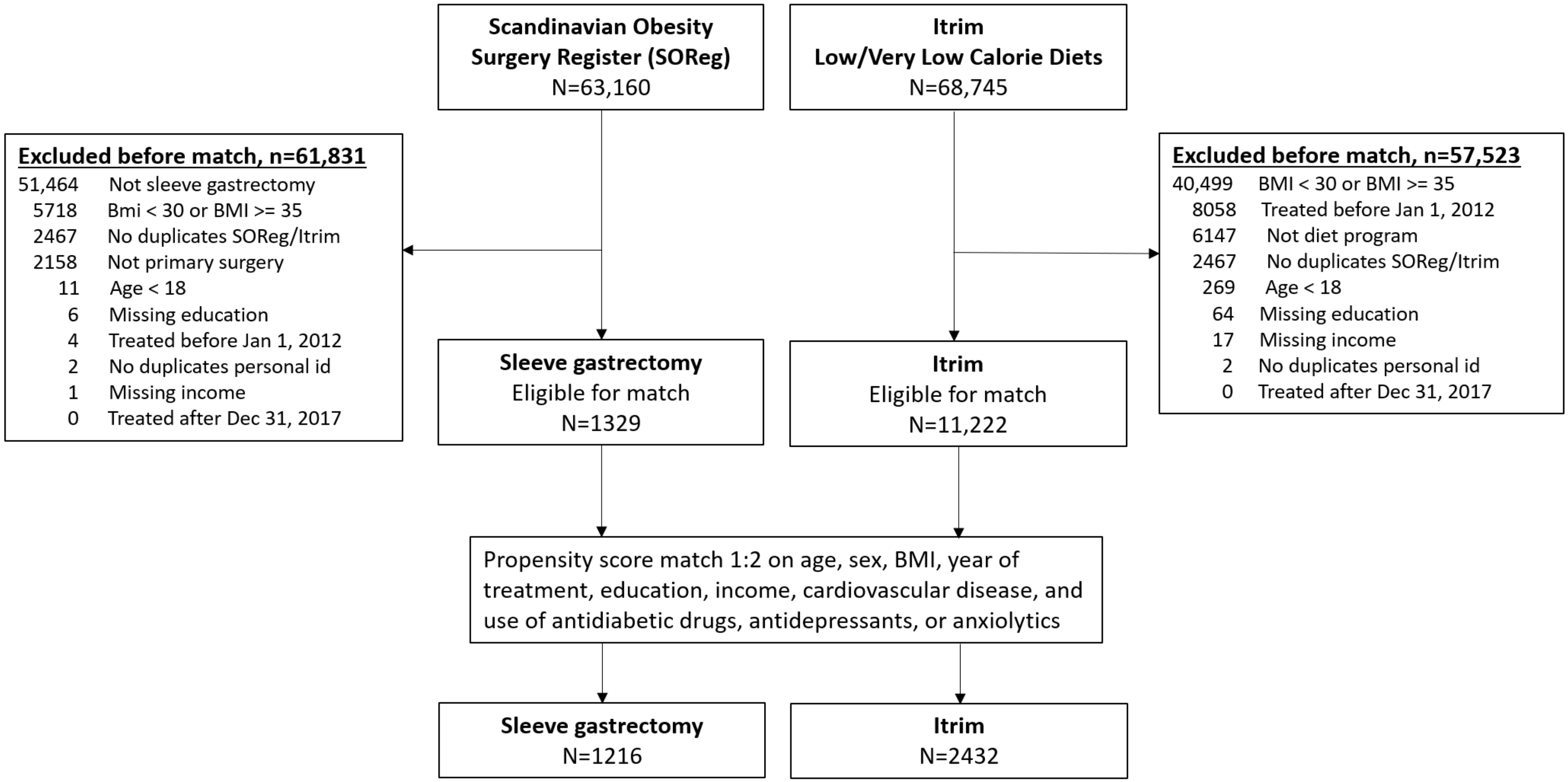

eFigure 1 Flowchart (sequential exclusions)

© 2022 Stenberg E et al. JAMA Network Open.

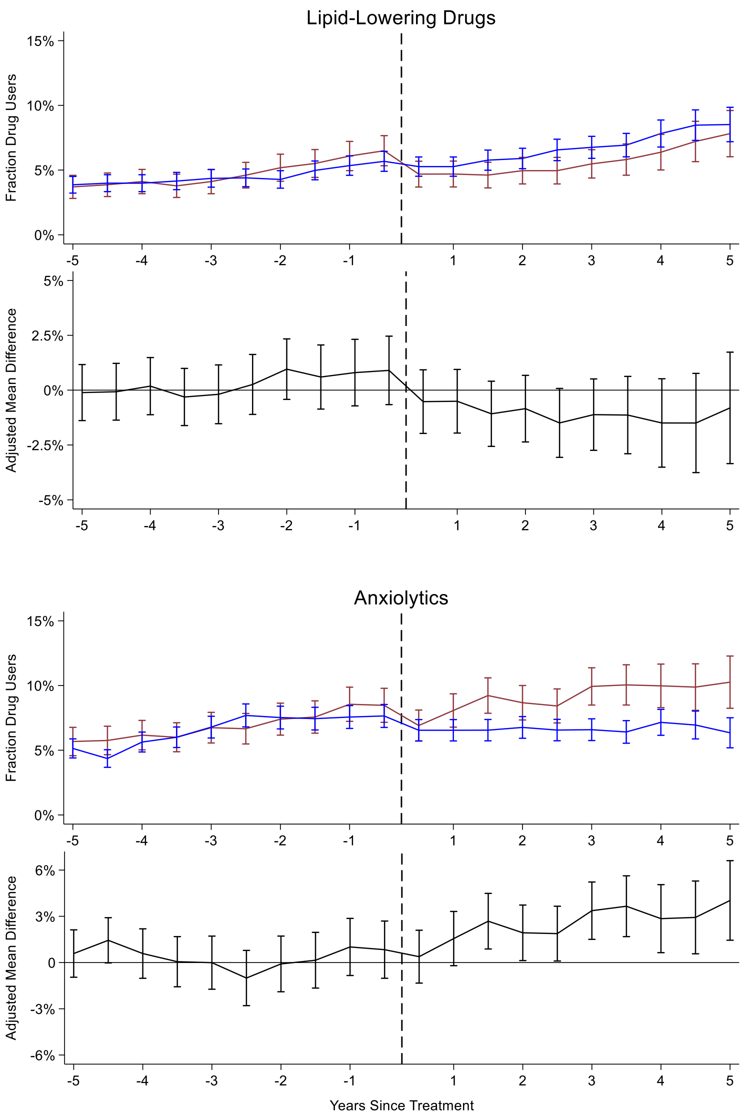

eFigure 2 Use of lipid-lowering drugs and anxiolytics. Red representing surgery group, blue representing intensive lifestyle modification group

© 2022 Stenberg E et al. JAMA Network Open.

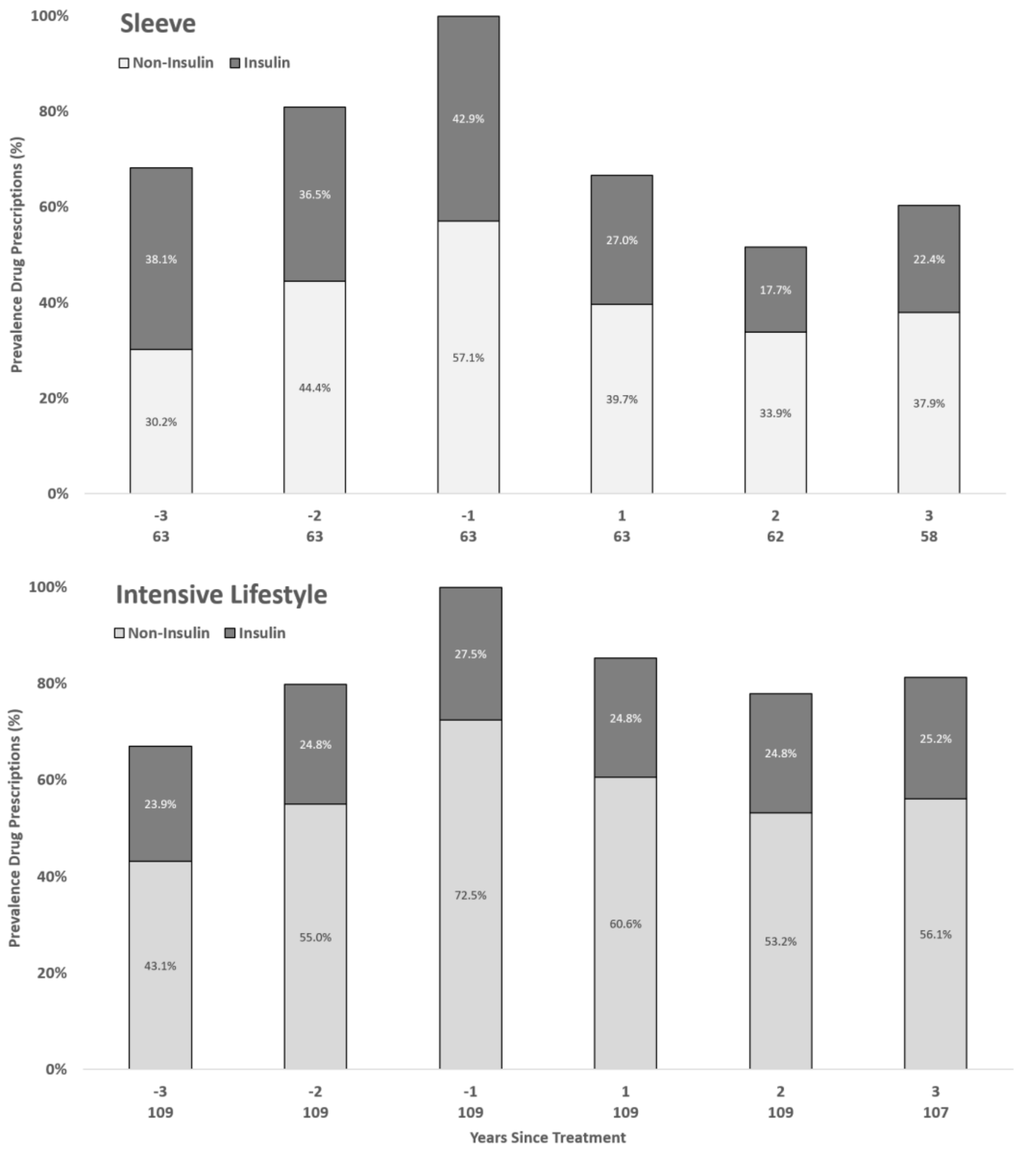

eFigure 3 Diabetes medication before and after treatment for patients with diabetes drug treatment before intervention

© 2022 Stenberg E et al. JAMA Network Open.

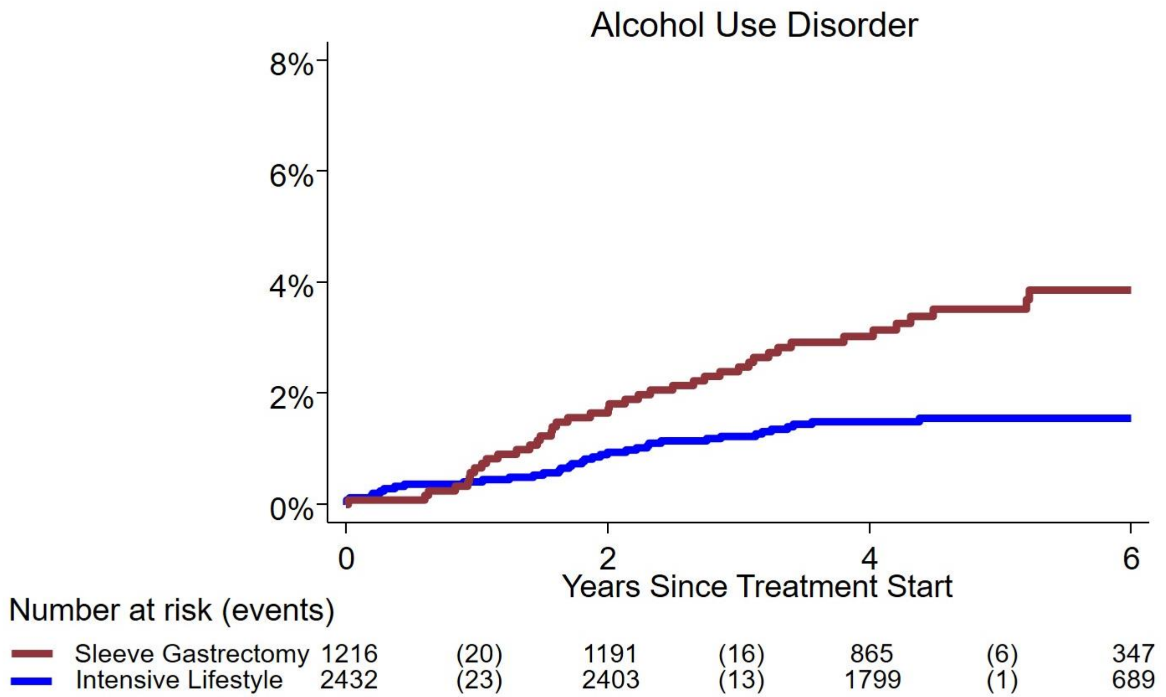

eFigure 4 Alcohol use disorders after intervention for patients receiving sleeve gastrectomy or intensive lifestyle treatment showing higher incidence of alcohol use disorders among patients receiving sleeve gastrectomy (69 vs 30 per 10,000 person-years; HR 2.24, 95%CI 1.45-3.48; P<0.001)

© 2022 Stenberg E et al. JAMA Network Open.

eTable 1 Outcome definitions

Outcomes	Data Source	ICD/ATC Codes	Comment
1y weight & BMI loss	SOReg & Itrim	Not applicable	
Diabetes drug use	Prescribed Drug Register	A10	
Antihypertensive therapy	Prescribed Drug Register	C02, C03A, C03B, C03EA01, C07 (except C07AA07), C08C, C08G, C09	
Inpatient CVD care	National Patient Register	I00-I99	
**SAFETY**			
Any complication 30d	SOReg	-	
Serious complication 30d	SOReg	-	
Revision surgery	SOReg	-	
Any admission<br>*(first 30 days after index date)*	National Patient Register	Any entry	
Gastric acid suppressing agents	Prescribed Drug Register	A02BC, A02BA	
Inpatient psychiatric care	National Patient Register	F00-F99	
Substance use disorder	National Patient Register<br>Prescribed Drug Register	F10-F19<br>N07BB, N07BC	Out- & inpatient care
Suicide<br>or self-harm	Causes of Death Register<br>National Patient Register	X60-84, Y10-Y34, Y87.0	Main & contributing causes<br>Out- & inpatient care
Antidepressants	Prescribed Drug Register	N06A	
Anxiolytics	Prescribed Drug Register	N05B	

© 2022 Stenberg E et al. JAMA Network Open.

eTable 2 Description and definitions of propensity score variables

Variable	Categories & Definition	Data Source	Comment
Age	Continuous	Total Population Register	
Sex	Woman/Man (Biological sex)	Total Population Register	
BMI	Continuous	Height and weight from SOReg & Itrim	
Treatment year	2012, 2013, 2014, 2015, 2016, 2017	SOReg operation date<br>Itrim program start date	
**Socioeconomic**			
Education level	Highest attained (<10y/10-12/>12y)	LISA Register	<0.6% missing
Disposable income	Mean of last 2y<br>Adjusted to 2016 price level, converted to USD with exchange rate from December 30, 2016	LISA Register	<0.2% missing
**Comorbidities**			
Diabetes	Diabetes drug treatment	Prescribed drugs register	ATC A10
CVD history	In- or outpatient admission	National Patients register	ICD10 chapter "I"
**Psychiatric**			
Antidepressant use		Prescribed drugs register	ATC N06A
Anxiolytics		Prescribed drugs register	ATC N05B

© 2022 Stenberg E et al. JAMA Network Open.

eTable 3 Surgical complications within 30 days following sleeve gastrectomy

COMPLICATIONS	BMI 30-<35				BMI ≥ 35			
	Yes	No	Missing (n)	%	Yes	No	Missing (n)	%
Readmission 30 Days, n (%)	33	1183	0	2.7	243	6168	0	3.8
Serious Complication[1]	16	1090	110	1.4	115	6079	217	1.9
Postoperative Complication	56	1050	110	5.1	347	5847	217	5.6
Leak	4	1102	110	0.36	35	6159	217	0.57
Bleeding	9	1097	110	0.81	73	6121	217	1.18
Abcesses/Deep Intraabdominal Infection	3	1103	110	0.27	22	6172	217	0.36
Abdominal Wall Complication	11	1095	110	0.99	67	6127	217	1.08
Bowel Obstruction	0	1106	111	0	8	6186	216	0.13
Stricture	2	1104	111	0.18	11	6183	216	0.18
Marginal Ulcers	3	1103	110	0.27	2	6192	217	0.03
Cardiovascular Complication	2	1104	110	0.18	2	6192	217	0.03
DVT/PE	1	1105	110	0.09	3	6191	217	0.05
Pulmonary Complication	7	1099	110	0.63	16	6178	217	0.26
Urinary Tract Infection	6	1100	110	0.54	26	6168	217	0.42
Other Complication	22	1084	110	1.99	148	6046	217	2.39

[1] Clavien-Dindo grades 3b, 4a, 4b or 5

© 2022 Stenberg E et al. JAMA Network Open.
